# Evolutionary conservation and metabolic significance of autophagy in algae

**DOI:** 10.1098/rstb.2023.0368

**Published:** 2024-09-30

**Authors:** Juliette Laude, Matteo Scarsini, Charlotte Nef, Chris Bowler

**Affiliations:** ^1^ Institut de Biologie de l’École Normale Supérieure (IBENS), École Normale Supérieure, CNRS, INSERM, PSL Université Paris, Paris 75005, France; ^2^ Université Paris Saclay, Gif-sur-Yvette 91190, France

**Keywords:** autophagy, ATG, algae, phytoplankton, stress, metabolism

## Abstract

Autophagy is a highly conserved ‘self-digesting’ mechanism used in eukaryotes to degrade and recycle cellular components by enclosing them in a double membrane compartment and delivering them to lytic organelles (lysosomes or vacuoles). Extensive studies in plants have revealed how autophagy is intricately linked to essential aspects of metabolism and growth, in both normal and stress conditions, including cellular and organelle homeostasis, nutrient recycling, development, responses to biotic and abiotic stresses, senescence and cell death. However, knowledge regarding autophagic processes in other photosynthetic organisms remains limited. In this review, we attempt to summarize the current understanding of autophagy in algae from a metabolic, molecular and evolutionary perspective. We focus on the composition and conservation of the autophagy molecular machinery in eukaryotes and discuss the role of autophagy in metabolic regulation, cellular homeostasis and stress adaptation in algae.

This article is part of the theme issue ‘The evolution of plant metabolism’.

## 1. Introduction

Proteins and organelles orchestrating cellular metabolism are perpetually maintained in a balanced cycle of biosynthesis and degradation. Two highly conserved processes are in charge of the degradation and recycling of intracellular components in eukaryotes: the ubiquitin-proteasome degradation system, which selectively removes damaged or unnecessary proteins, and autophagy, a ‘self-digesting’ pathway.

The first observations of bulk sequestration and digestion of intracellular components in membrane structures were reported for animal tissues in the 1950–1960s (reviewed in [1]). This new phenomenon, i.e. the delivery of cytoplasmic elements to lysosomes for degradation, was coined ‘autophagy’ in 1963 [2]. Since then, the presence of an autophagic activity as well as genes essential for this process have been documented in metazoans, fungi, plants and several algal groups. The modern definition of autophagy now refers to a group of diverse processes mediating the transport of endogenous cytoplasmic components (cytosol, macromolecules, protein complexes and aggregates, organelles, etc.) as well as exogenous entities (bacteria, viruses or parasites) to lytic organelles (lysosomes in animals or vacuole in fungi and plants) for degradation and recycling [3].

**Figure 1.**
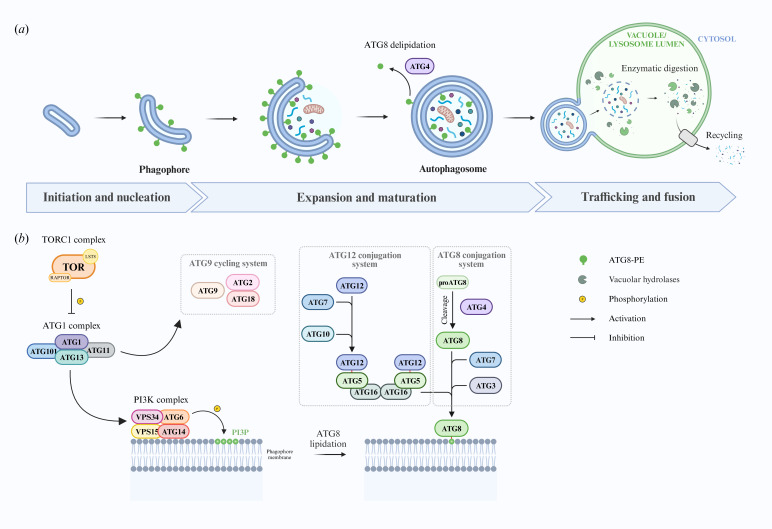
Cytological and molecular events of macroautophagy. Created with BioRender.com. (*a*) The induction of macroautophagy triggers the formation and expansion of a cup-shaped small membrane sac called the phagophore at the phagophore assembly site (PAS). The expanding phagophore membrane bends and eventually seals itself into an autophagosome, capturing a portion of the cytoplasm containing the cargo. Once closed off, the autophagosome is trafficked to the lytic organelle. Upon delivery, the outer membrane of the autophagosome fuses with that of the lytic compartment. Breakdown of its inner membrane finally exposes its contents to hydrolases, which degrade it into basic metabolic building blocks (i.e. amino acids, nucleotides, sugars and fatty acids) to be exported back to the cytosol for reuse. (*b*) The initiation of autophagy is regulated at the ATG1 kinase complex by developmental and nutritional cues. ATG13 dephosphorylation in autophagy-promoting conditions allows it to bind and activate ATG1. The activated serine/threonine kinase ATG1 promotes several key downstream autophagy steps. Membrane lipids are delivered to the PAS thanks to the activation of the transmembrane protein ATG9 and its cycling partners ATG2 and ATG18, promoting the nucleation and expansion of the phagophore. The forming phagophore membrane is decorated with phosphatidylinositol-3-phosphate (PI3P) by the PI3K complex, promoting vesicle nucleation and the recruitment of other ATG proteins to the PAS. PI3P decoration allows for the conjugation of ATG8 to phosphatidylethanolamine (PE) via a conjugation pathway analogous to ubiquitylation, the Ubl conjugation system. The ATG4 protease cleaves the Ub-like protein ATG8 inactive precursor (proATG8), exposing a conserved C-terminal glycine residue. ATG8 is activated by the E1 and E2 conjugating enzymes ATG7 and ATG3, to be finally covalently linked to PE by the action of the ATG5-ATG12-ATG16 E3 ligase complex. Lipidated ATG8 (ATG8-PE) promotes the elongation, sealing and later fusion of the autophagosome with the lytic organelle in yeast and mammals [4]. ATG8-PE decorations tethered to the outer autophagosome membrane are eventually delipidated by ATG4 for reuse.

Autophagic processes are distinguished based on their degree of selectiveness, their targets (referred to as ‘cargos’) and the type of sequestration mechanism involved. The best characterized and most conserved autophagic pathway is macroautophagy (figure 1*a*), which involves the sequestration of cargos within double membrane cytosolic vesicles named autophagosomes, and their transport to a lytic organelle for degradation. The cytological features of macroautophagy are very similar between eukaryotic groups as distant as opisthokonts and embryophytes, though a detailed analysis reveals significant ultrastructural and regulatory differences [5–7]. The protein machinery is encoded by the conserved autophagy-related (ATG) genes as well as a large number of lineage-specific actors recruited through independent evolution in the different eukaryotic lineages. In addition to macroautophagy, different alternative autophagic pathways have been described. Observed in animals, yeast and plants [8], microautophagy does not rely on the de novo formation of a sequestering compartment. Instead, cargos are captured and engulfed directly into the lysosome or the vacuole by invagination or protrusion of its membrane. Despite the lack of autophagosome formation, part of the core macroautophagy machinery is directly involved in microautophagy. Moreover, distant eukaryotic groups appear to have evolved lineage-specific autophagic pathways, namely, chaperone-mediated autophagy in opisthokonts [9] and mega-autophagy in plants [10].

In the absence of stress, autophagy functions as a cellular housekeeper, clearing away aged, damaged or unnecessary cytoplasmic components, thus participating in the maintenance of cellular homeostasis. As an example, the maintenance of a healthy population of organelles (e.g. mitochondria, chloroplasts and peroxisomes) is essential for cellular homeostasis and relies, among other things, on autophagy [11–15]. Accumulation of dysfunctional mitochondria leads to a shortened lifespan in longevity-extending conditions, reactive oxygen species (ROS) accumulation and mtDNA damage in yeast [16,17]. In animal models, loss of mitophagy promotes increased sensitivity to various tissue injuries, neurodegeneration, tumorigenesis, inflammation and ageing [18]. Moreover, autophagic genes are generally upregulated in response to environmental fluctuations, such as nutrient scarcity and oxidative stress [19–21], and promote stress tolerance by recycling cytoplasmic material into metabolites for the synthesis of essential components and as an energy supply [22–29]. Other functions of autophagy include endoplasmic reticulum stress management [30–32], pathogen removal [33–35] and cellular or metabolic remodelling during cell differentiation and development [36–38].

Algae are a diverse group of photosynthetic eukaryotes that play crucial roles in aquatic ecosystems, acting as primary producers in freshwater and marine environments and constituting the base of aquatic food webs. Marine algae account for roughly 12% of oceanic biomass (0.8 Gt C) [39] and, in concert with photosynthetic cyanobacteria, perform almost as well as land plants, which represent around 80% of terrestrial biomass (450 Gt C) [40] in terms of carbon fixation [41]. Algae can be found in a multitude of forms, including single-celled microorganisms and larger multicellular seaweeds. Modern algae groups belong to several evolutionarily distant lineages but are connected through the acquisition of the chloroplast via several independent primary, secondary or tertiary endosymbiotic events [42]. While mounting evidence points to an important role of autophagy in several key aspects of algal life, including acclimation to environmental stress and lipid metabolism, very little attention has been paid to the mechanisms involved. In this review, we summarize the current knowledge of autophagy in algae from a metabolic, molecular and evolutionary perspective. We focus on the composition and conservation of the autophagy molecular machinery and discuss the role of autophagy in metabolic regulation, cellular homeostasis and stress responses. Finally, we propose future promising areas of investigation into autophagic pathways in algae.

## 2. Autophagy in algae

### (a) Experimental evidence for autophagy in algae

Direct observation of the presence of autophagic-like vesicles and vacuoles has been reported in a few evolutionarily distant algal lineages (table 1). Sequestration and apparent degradation of cytoplasmic components inside vesicular or vacuolar structures either formed de novo or through tonoplast invagination was reported in the brown algae *Ectocarpus siliculosus* and *Cystoseira stricta* long before the advent of genomic studies [82,83]. Both studies demonstrated the presence of acid phosphatases in these autophagic-like vacuolar structures, likely mediating the degradation of their content. Other instances of autophagic-like vesicles were captured in Stramenopile algae, such as in the diatom *Cyclotella meneghiniana* [55,71], in the Eustigmatophyceae *Nannochloropsis oceanica* [52], and in the giant kelp *Macrocystis pyrifera* [80]. In the haptophyte *Emiliania huxleyi*, autophagosome-like double-membrane vesicles appeared in cells in response to viral infection [79]. Autophagic processes have generally been more extensively studied in green algae, such as in *Dunaliella primolecta* [53], *Desmidium swartzii* [70], *Micrasterias denticulata* [44,64,69], *Chlamydomonas reinhardtii* [43,47,72,84,85], *Lobosphaera incisa* [49] and *Auxenochlorella protothecoides* [81].

**Table 1.** Experimental evidence of autophagy induction under different abiotic and biotic stresses in algae. TEM, transmissionelectron microscopy. MDC (monodansylcadaverine) and Lysotracker both stain acidic compartments and are indicative of a degradative process within the cells.

stress condition	species	references	evidence of autophagy	evidence of ROS accumulation ?
**nutrient limitation**				
carbon starvation	*Chlamydomonas reinhardtii*	[43]	ATG8 protein accumulation and lipidation	
	*Micrasterias denticulata*	[44]	TEM observation of autophagosomes	[45]
nitrogen starvation	*Chlamydomonas reinhardtii*	[43,46]	ATG8 protein accumulation and lipidation	
		[47]	TEM observation of vacuoles/autophagosomes	
			upregulation of ATG gene expression	
		[48]	ATG8 and ATG3 protein accumulation	
	*Lobosphaera incisa*	[49]	ATG8 protein lipidation	
			TEM observation of autophagosomes	
			upregulation of ATG gene expression	
	*Phaeodactylum tricornutum*	[50,51]	upregulation of ATG gene expression	
	*Nannochloropsis oceanica*	[52]	ATG8 protein lipidation	
			upregulation of ATG gene expression	
nutrient limitation (stationary phase)	*Dunaliella primolecta*	[53]	TEM observation of autophagosomes	
	*Chlamydomonas reinhardtii*	[43]	ATG8 protein accumulation and lipidation	
sulfur limitation	*Chlamydomonas reinhardtii*	[45]	upregulation of ATG8 gene expression	
phosphate limitation	*Chlamydomonas reinhardtii*	[46,54]	ATG8 protein accumulation and lipidation	
	*Cyclotella meneghiniana*	[55]	TEM observation of autophagosomes	
	*Prorocentrum shikokuense*	[56]	MDC+bodies	
			upregulation of ATG gene expression	
	*Emiliania huxleyi*	[57]	ATG8 protein lipidation	
			TEM observation of autophagic vacuoles	
			MDC+bodies, Lysotracker+bodies	
nutrient limitation (polar night)	*Fragilariopsis cylindrus*	[58]	upregulation of ATG gene expression	
**other abiotic stress**				
high light stress	*Chlamydomonas reinhardtii*	[59–61]	ATG8 protein accumulation and lipidation, ATG3 protein accumulation	[62]
			ATG8+bodies	NO burst [63]
high salinity	*Micrasterias denticulate*	[64]	TEM observation of autophagosomes	[52]
	*Haematococcus lacustris*	[65]	TEM observation of autophagosomes	
			ATG8+bodies, MDC+bodies	
	*Ectocarpus siliculosus*	[66]	upregulation of ATG8 and ATG9 gene expression	
excess nickel, copper and cobalt ions	*Chlamydomonas reinhardtii*	[67]	ATG8 protein accumulation and lipidation	
			ATG8+bodies	
			upregulation of ATG8 gene expression	
copper stress	*Ectocarpus siliculosus*	[68]	upregulation of ATG8 gene expression	
chromium stress	*Cyclotella meneghiniana*	[55]	TEM observation of autophagosomes	
cadmium stress	*Micrasterias denticulate*	[69]	TEM observation of autophagosomes	[53]
	*Desmidium swartzii*	[70]	TEM observation of autophagosomes	
chlorinated benzenes	*Cyclotella meneghiniana*	[71]	TEM observation of autophagosomes	
oxidative stress induced by methyl violet	*Chlamydomonas reinhardtii*	[59,60]	ATG8 protein accumulation and lipidation, ATG3 protein accumulation	[62]
			ATG8+bodies	
oxidative stress by deficiency in photoprotective pigments	*Chlamydomonas reinhardtii*	[59,60,72]	ATG8 protein accumulation and lipidation, ATG3 protein accumulation	[62]
			ATG8+bodies	
oxidative stress induced by H_2_O_2_	*Chlamydomonas reinhardtii*	[43,59,73]	ATG8 protein accumulation and lipidation	by definition
	*Ectocarpus siliculosus*	[66]	upregulation of ATG8 and ATG9 gene expression	by definition
**protein quality stress**				
ClpP-repression induced chloroplast protein quality control stress	*Chlamydomonas reinhardtii*	[74]	ATG8 protein accumulation and lipidation, ATG3 protein accumulation	
			ATG8+bodies	
			upregulation of ATG gene expression	
ER stress	*Chlamydomonas reinhardtii*	[43,48,67,75]	ATG8 protein accumulation and lipidation	[76]
**organelle integrity stress**				
loss of chloroplast membrane integrity	*Chlamydomonas reinhardtii*	[77]	ATG8 protein accumulation and lipidation	[78]
**biotic stress**				
viral infection	*Emiliania huxleyi*	[79]	ATG8 protein lipidation	
			TEM observation of autophagosomes	
			MDC+bodies, Lysotracker+bodies	
			Upregulation of ATG gene expression	
oomycete infection	*Macrocystis pyrifera*	[80]	TEM observation of autophagic vacuoles	
			MDC+bodies	
**metabolic/developmental transitions**				
heterotroph-to-autotroph (HA) transition	*Auxenochlorella protothecoides*	[81]	TEM observation of autophagic vacuoles	
			MDC+bodies	

These evolutionarily distant algal lineages appear to display the fundamental hallmarks of autophagy described in yeast, plants and metazoans, i.e. the formation of sequestration compartments undergoing acidification for the degradation of their content. Autophagosomes and autophagic vacuoles were observed mostly in stress conditions and rarely in optimal growth conditions. Double membrane autophagosomes were clearly observed in *E. siliculosus* and *C. stricta* [82,83], in *E. huxleyi* [79] and in the green algae *D. primolecta* [53] and *C. reinhardtii* [85]. However, most autophagic-like compartments observed in algae were delimited by a single membrane, indicating that they were vacuoles. It is not clear whether these represent a later stage in the autophagic process (i.e. the fusion of an autophagosome with a vacuole), or if they are the result of the direct engulfment of cargo inside a vacuole (i.e. microautophagy), which has been reported in the brown macroalgae *Ectocarpus* and *Macrocystis* [80,82] and potentially in green algae [49,86]. Taken together, these observations suggest that both macroautophagy and microautophagy might be conserved at least between metazoans, Archaeplastida, stramenopiles and haptophytes. Moreover, the nature of the cargos recycled through autophagy in algae has not yet been thoroughly addressed. TEM analyses showed autophagosomes and autophagic vacuoles containing cytoplasmic components, membranous materials [47,49], mitochondria [44,53], chloroplasts [44,80], lipid droplets (LDs) [44,49,52,81,86] or pathogens [80].

### (b) Autophagy-related genes: evolutionary conservation and functional significance in algae

Direct experimental evidence of autophagic processes in algae are still very limited and concern only a handful of species that do not encompass all algal lineages. To date, the most frequently applied method to identify autophagic processes in algae is to check for the presence of core autophagy genes within genomes.

#### (i) Conservation of the core autophagy machinery

Autophagic processes are mediated by the highly conserved ATG proteins. First identified and characterized in *S. cerevisiae*, particularly thanks to the pioneering genetic screens developed in the lab of Yoshinori Ohsumi, it later became clear that most yeast ATG genes had orthologues in mammal and plant model species as well [3,6]. In addition to numerous lineage-specific essential and accessory actors [3], more than 40 ATG proteins have been described, partaking in various autophagy pathways [87]. Nineteen of these constitute what is termed the ‘core autophagy machinery’, which is essential for autophagosome formation, and can be divided into four functional units: the ATG1 kinase complex (ATG1/ULK1, ATG13, ATG11 and ATG101), the ATG9 cycling system (ATG9, ATG2 and ATG18), the phosphatidylinositol 3-kinase (PI3K) complex (VPS34, ATG6/VPS30/BECN1, ATG14 and VPS15) and the ubiquitin-like protein (Ubl) conjugation system (ATG8, ATG12, ATG3, ATG10, ATG7, ATG4, ATG5 and ATG16) (figure 1*b*). While these actors are required for macroautophagy, some are also involved in microautophagic processes.

**Figure 2.**
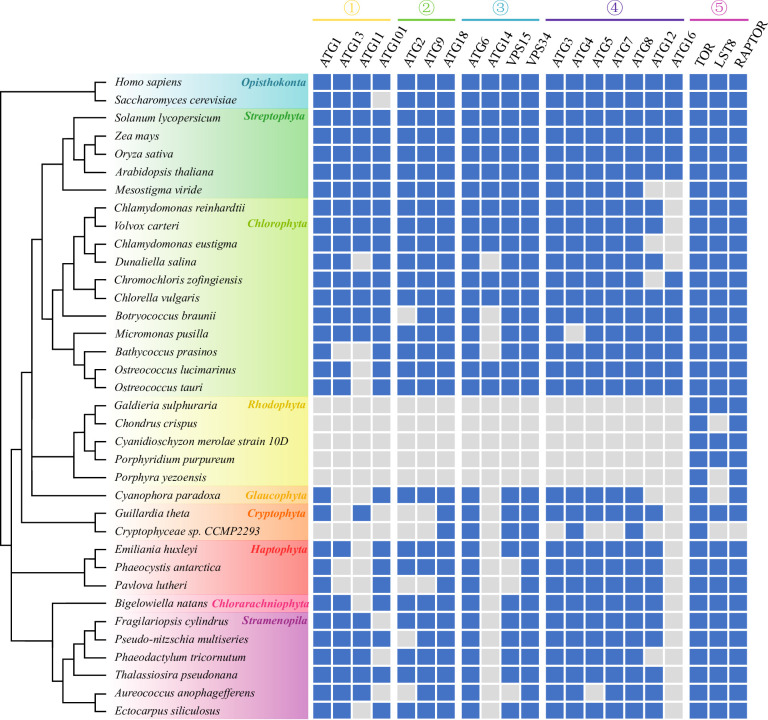
Conservation of core autophagy genes and TORC1 complex components in different eukaryotic lineages (1). ATG1 kinase complex (2), ATG9 cycling system (3), PI3K complex (5), Ubl conjugation system [6], TORC1 kinase complex. Branch lengths of the cladogram are non-informative. Adapted from Mallén-Ponce *et al.* [90,91]. ATG11 and ATG101 protein sequences were identified in algal genomes by BLASTP search using *H. sapiens*, *S. cerevisiae* and *A. thaliana* input sequences and validated by reciprocal BLASTP against the NCBI protein database. Representative sequences of each protein are available in electronic supplementary material, table S1 (sequence IDs are derived from [90,91] and this study).

Taking advantage of the recent availability of numerous algal genomes, several authors have explored the conservation of ATG proteins in representatives of most photosynthetic eukaryotes (Chlorophyta, Rhodophyta, Glaucophyta, Cryptophyta, Haptophyta, Chlorarachniophyta and Ochrophyta (photosynthetic stramenopiles)) [88–90], with the exception of photosynthetic alveolates (dinoflagellates; although the presence of several *ATG* genes has been reported in *Prorocentrum shikokuense* [56]) and excavates (euglenids). Most core autophagy genes previously defined in yeast, plants and mammals were identified in the algal lineages surveyed (figure 2) except for red algae. Mounting genomic evidence indicates that ATG proteins are a highly conserved eukaryotic feature [92–95]. Thus, autophagy might have emerged during the process of eukaryogenesis and have been present in the last eukaryotic common ancestor (LECA) [96].

Fifteen core ATG genes (*ATG1*, *ATG13*, *ATG101*, *ATG6*, *VPS15*, *VPS34*, *ATG9*, *ATG2*, *ATG18*, *ATG5*, *ATG3*, *ATG4*, *ATG7*, *ATG8* and *ATG12*) are present in most algal lineages [90] (figure 2), while *ATG14* and *ATG16* appear to be absent from all non-Archaeplastida algal genomes surveyed to date. Single *ATG* orthologs were missing in certain algal genomes while other gene products participating in the same functional unit of the autophagic machinery were highly conserved. In particular, very few orthologs of genes encoding the core ATG proteins were uncovered in the Cryptophyta Cryptophyceae sp. CCMP2293 and in the Haptophyta *Phaeocystis antarctica* and *Pavlova lutheri* genomes. We cannot exclude the possibility that these genes could not be uncovered by *in silico* analysis because of high sequence divergence or incomplete genomes, algal genomes being for the most part recent with heterogeneous assembly quality and genome completeness [97]. It could also suggest that some core ATG proteins may be dispensable in certain species or have been functionally substituted by lineage-specific functional analogs. As sequence similarity does not equate conservation of function, experimental studies are required to verify that algal *ATG* genes predicted from *in silico* and mRNA analysis do indeed perform similar functions to that of their known orthologs.

Contrary to closely related plants and green algae, no orthologs of the core *ATG* genes could be identified in genomes from unicellular and multicellular red algae [89,90]. Comparative genomics of Rhodophyta genomes strongly suggests that the common ancestor of red algae underwent genome reduction, possibly by adaptation to a very restrictive ecological niche [98]. Mallén-Ponce *et al.* [90] have hypothesized that the uniformity of this putative restrictive niche may have limited the need for recycling processes in the red algal common ancestor, causing the loss of cellular functions such as autophagy under relaxed selective pressure. However, the authors also noted that vacuole-like structures were reported in *Cyanidioschyzon merolae* [99,100], which suggest that ATG-independent mechanisms to target substrates to vacuoles for degradation do exist in at least some Rhodophytes.

#### (ii) Mechanistic insights in algae

After two decades of mechanistic studies in various model organisms, a highly conserved mechanism for autophagosome formation has emerged (reviewed in [6,101,102]; figure 1*b*). What do we know about macroautophagy mechanisms in algae? Modelling of ATG4, ATG8, ATG3 and ATG7 three-dimensional structures revealed a high degree of structural conservation between evolutionarily distant photosynthetic organisms and classical biological models [90]. Several residues with critical functions in autophagosome formation in yeast, animals and plants are conserved in the catalytic and binding sites of algal ATG proteins. For instance, the multimerization of yeast and human ATG9 proteins via their conserved L^766^GYVC^770^ and V^515^GDTC^519^ respective motifs is essential for membrane flow to the expanding phagophore [103,104]. These motifs are also present in plant and algal ATG9 orthologs with the consensus sequence VGXVC [89]. Similarly, algal ATG proteins involved in the Ubl conjugation system maintain key residues essential for ATG8 activation and conjugation [88,89]. During lipidation, ATG8 is covalently coupled to ATG7 and ATG3 and finally to PE at its highly conserved carboxy-terminal glycine (Gly116) in yeast, animals and plants [105–107]. This glycine residue is conserved in all algal ATG8 homologs surveyed [81,88,89] (electronic supplementary material, figure S1). On the contrary, the ATG8 C-terminal extension downstream of Gly116, which is cleaved by the ATG4 protease, is quite variable between and within species [89,95]: it is particularly large in certain species (e.g. *V. carteri* and *P. antarctica*) while being completely absent in others (e.g. *E. siliculosus* and *A. thaliana* ATG8h-i) (electronic supplementary material, figure S1), indicating that ATG8 could be conjugated to PE without ATG4-mediated cleavage. Taken together, the conservation of ATG3, ATG5, ATG7, ATG8, ATG10, ATG12 and ATG9 catalytic and binding sites support the assumption that they assume similar functions in autophagosome formation in algae compared to their yeast, animal and plant counterparts.

Most functional and mechanistic data regarding autophagic processes currently available in algae are derived almost exclusively from the study of the green algal model *C. reinhardtii*. Pérez-Pérez *et al.* [43] showed that *C. reinhardtii* ATG8 (CrATG8) functionally complements yeast ATG8 in *atg8*Δ mutant cells. Nascent CrATG8 is cleaved by endogenous yeast Atg4 at the conserved Gly120 residue (equivalent to ScATG8 Gly116), a step required for functional complementation. Following in their footsteps, Zhao *et al.* [81] showed that *A. protothecoides* ATG8 is also cleaved specifically at its conserved glycine residue Gly118 by ScAtg4 when expressed in yeast *atg8*Δ cells. ApATG8 can be conjugated *in vitro* to PE by the yeast Ubl conjugation system and is localized to the PAS during induction of autophagy, as is ScAtg8 when expressed in yeast *atg8*Δ. Conversely, ApATG4 can functionally replace ScAtg4, restoring the processing of endogenous Atg8 and its translocation to the vacuole under nitrogen (N) starvation in yeast *atg4*Δ mutants.

Subsequent work has taken advantage of the numerous genetic tools available in *C. reinhardtii* to explore the mechanisms of autophagy closer to natural conditions. It was shown that CrATG8 is processed shortly after synthesis into its shorter mature form by CrATG4 [43,108]. Mature CrATG8 accumulates and is lipidated under autophagy-activating conditions, such as nutrient limitation, H_2_O_2_-induced oxidative stress and TOR inhibition [43]. ATG8 lipidation into ATG8-PE was also demonstrated in the green alga *L. incisa* and in the stramenopile *N. oceanica* during N starvation [49,52], as well as in *E. huxleyi*, a coccolithophore belonging to the distant Haptophyta group, during autophagy induction by viral infection [79]. Return to optimal growth conditions after nutrient limitation resulted in the rapid delipidation and decrease in abundance of CrATG8 [43], showing that the process is reversible and probably involves the activity of ATG4. Moreover, ATG3 is required for ATG8 lipidation in *C. reinhardtii* [48]. Finally, pharmacological inhibition of PI3K complex activity blocks the formation of autophagic vacuoles in *Chlorella variabilis* [88] and the lipidation of ATG8 in *E. huxleyi* [79], suggesting that, like in yeast, animals and plants, the activation of the PI3K complex is required for subsequent ATG8 conjugation and autophagosome formation in these distant algal lineages. Several key features of the ATG8 conjugation machinery appear to be conserved in algae, but the molecular characterization of autophagy mechanisms is still in its infancy and lacks more molecular insights derived from a varied panel of algal lineages.

#### (iii) On the selectiveness of autophagy in algae

Autophagy was long thought to be a non-selective degradation mechanism, digesting portions of the cytoplasm indiscriminately. However, it has now become clear that it can target cargo selectively, due to specific receptors that recognize and tether cargo to the expanding phagophore membrane [6,76,109,110]. Selective loading of cargo into the forming phagophore is ensured by the interaction of their respective receptors with lipidated ATG8. Receptors bind to ATG8 either through an ATG8-interacting motif (AIM) (or LC3-interacting region (LIR) in animals) which recognizes the ATG8 AIM-docking site [110] or through an ubiquitin-interacting motif (UIM) that recognizes the ATG8 UIM-docking site [111] in yeast, animals and plants. Although little attention has been paid to the nature of the cargos engulfed in autophagosomes and vacuoles, several lines of evidence suggest that autophagy works selectively in algae too. AIM- and UIM-docking sites both show a high degree of conservation in algal ATG8 proteins [90] (electronic supplementary material, figure S1). Moreover, ATG8 was shown to directly interact with the lipid droplet surface protein LDSP through its AIM motif in *N. oceanica*, probably mediating the specific engulfment of lipid droplets into forming autophagosomes [52]. Several known autophagy receptors for pexophagy and mitophagy identified in yeast are absent in algal and plant genomes [78,88]; however, it is likely that a significant number of cargo receptors are lineage-specific. In contrast, the evolutionarily conserved autophagy receptor NBR1, which was shown to mediate autophagic removal of a large panel of cargos including protein aggregates and peroxisomes in yeast, animals and plants, is encoded within algal genomes [112–114]. Whether NBR1 also functions as a selective autophagy receptor in algae remains to be addressed.

## 3. Physiological roles of autophagy in algae

### (a) Survival and adaptation to stress

The induction of autophagy in response to a large panel of abiotic and biotic stressors has been extensively documented in yeast, animals and plants [22,23,115]. Autophagy was shown to promote tolerance and adaptation through nutrient recycling, though it may promote or even mediate cell death in certain instances [116]. Likewise, there is a growing body of microscopic, proteomic and transcriptomic evidence showing that autophagic responses are triggered by a wide range of stress conditions in algae (table 1).

#### (i) Nutrient limitation

The upregulation of autophagy appears to be a common response to nutrient scarcity in algae (table 1). As an example, N limitation increases *C. reinhardtii* autophagic flux in less than 24 h, as measured by the accumulation and lipidation of CrATG8 *in vivo* [43]. Whereas CrATG8 is localized to a single spot in exponentially growing cells, autophagy induction during N limitation resulted in the multiplication of CrATG8 spots all over the cell, probably reflecting the active formation of mature autophagosomes [43,48]. Similarly, autophagy is activated in N- and phosphorus (P)-limiting conditions in other algal lineages [50–52,56,57]. This activation can be fully reversed by resupplying nutrients [43,52], showing how autophagy is finely tuned to cell nutrient status. Conversely, mutation of key autophagy genes *CrATG3* and *CrATG8* causes sensitivity to N, P and S starvation [48], indicating that autophagy is crucial for acclimation to multiple nutrient stresses in the green algae model, as previously reported in yeast [117] and plants [118–120].

Autophagy may promote cell survival by mitigating some of the negative effects of nutrient starvation through the remobilization and recycling of amino acids, lipids and carbohydrates derived from the degradation of damaged or unnecessary cytoplasmic components and organelles. Indeed, in N-starved yeast, autophagic recycling of amino acids is necessary to maintain protein synthesis and consequently a ROS scavenging activity and a healthy mitochondria pool [24,25]. Studies in mice and *Arabidopsis thaliana atg* mutants have highlighted how autophagy actively remodels metabolism and allocation of intracellular carbohydrates, amino acids and lipids to cope with nutrient limitation [26–28], for example, by providing a supply of alternative energy sources such as amino acids [26,29]. Interestingly, autophagy deficiency in *C. reinhardtii atg3* and *atg8* mutants had a major impact on the formation of starch and triacylglycerol (TAG) reserves under N, P and S starvation [48], suggesting that autophagy also plays a key role in stress-induced carbon reallocation in green algae, though the exact process remains to be explored.

#### (ii) Other abiotic stress

Algal autophagy is also induced in response to a large panel of other abiotic stressors, including excess metals, low and high salinity, high light and oxidative stress (table 1). Although metal ions are important for protein function in several key cellular processes, high concentrations can also have toxic effects and are common autophagy triggers [121]. Autophagy is required for salt tolerance in plants, by sequestering Na^+^ ions directly into the vacuole and removing damaged proteins [122]. In the green alga *Haematococcus lacustris*, boosting the autophagic flux during high salinity stress mitigates its negative effects on cell growth [65]. Under high light intensity, damaged chloroplasts and peroxisomes are selectively removed by autophagy in plants, limiting the accumulation of ROS and the advent of cell death [13,123].

Autophagy was strongly induced upon accumulation of misfolded proteins in the endoplasmic reticulum (ER) and chloroplast in *C. reinhardtii*, as observed in yeast and plant cells [30,31]. Indeed, treatment with tunicamycin, an antibiotic that raises the level of unfolded polypeptides in the ER by inhibiting protein glycosylation, or DTT, a strong reducing agent blocking disulfide-bond formation, strongly increased the abundance and lipidation of CrATG8 [43,48,67,75]. Likewise, inhibition of the activity of the ClpP complex, an essential chloroplast protease involved in protein quality control, triggered an autophagic response [74]. Cerulenin, an inhibitor of fatty acid de novo synthesis, triggers dramatic morphological changes in the chloroplast, loss of photosynthetic capacity and strongly activates autophagy in *C. reinhardtii* [77]. All these examples illustrate how autophagy could play a role in the disposal of misfolded proteins during ER and chloroplast unfolded protein response (UPR) and in organelle quality control in algae. However, it is also possible that autophagy is induced in the cytoplasm simply as a consequence of the cytotoxic effects of these stresses (i.e. ROS accumulation). Recent advances have established that degradation of ER portions is carried out by macro and microautophagy upon UPR induction, in concert with ER‐associated degradation (ERAD), and is essential to cellular homeostasis [32]. Likewise, autophagy directly removes specific portions or entire chloroplasts in plants, though its role in chloroplast UPR has not yet been established [124].

#### (iii) Pathogens

Hallmarks of intensive autophagy have been reported in two algal species challenged by pathogen infection. Digestive vacuoles containing pathogen material at different stages of degradation could be observed in the giant kelp *M. pyrifera* infected by the oomycete *Anisolpidium ectocarpii* [80]. Autophagy markers were also observed in the surrounding non-infected cells, suggesting that autophagy is part of the kelp systemic immune response. Moreover, autophagy-mediated plastid degradation was observed in both infected and non-infected cells. Therefore, autophagy may facilitate nutrient remobilization to sustain the local defence response during the host–pathogen arms race, in addition to direct pathogen degradation. Infection of *Emiliania* cells with the *E. huxleyi* virus (EhV), a giant double-stranded DNA coccolithovirus, was accompanied by the presence of double-membrane vesicles in the cytosol and the accumulation and lipidation of EhATG8 [79]. Interestingly, blocking lysosomal/vacuolar acidification did not impact viral production and extracellular release, suggesting that the viral particles were not digested. On the contrary, blocking autophagosome formation reduced the release of viral particles, while viral replication was not affected. Moreover, host ATG8-PE was found in purified virions. Overall, the host autophagy machinery appeared to be hijacked for virion production. Membranes originating from the host autophagic-like processes were incorporated into the newly formed viral structures before egress from the cells. As such, autophagy is likely to play a dual role in algae–pathogen interactions, as it can both serve as an intracellular host defence mechanism but can also be subverted by the pathogen to their advantage.

### (b) Lipid metabolism

Lipid homeostasis is essential for algal metabolism, especially upon exposure to stress conditions. In fact, a conserved response to nutrient starvation in algae is the remobilization of membrane lipids to synthesize energy storage TAGs, deposited in specialized cytosolic organelles called lipid droplets (LDs). These are composed of a core of neutral lipids, mostly TAGs, and delimited by a protein-associated polar lipid monolayer [125]. LDs represent reserves of energy, membrane components and carbon skeletons, as well as proteins; and as such are promptly consumed by microalgae upon nutrient resupply.

Autophagy selectively engulfs partial or complete LDs in autophagosomes or vacuoles for further degradation, a process that has been imaged in *M. denticulata* during carbon starvation [44], in *C. reinhardtii* [86], *L. incisa* [49] and *N. oceanica* [52] during recovery from N starvation or in *A. protothecoides* during the transition from heterotrophy to autotrophy [81]. These observations suggested that at least two different autophagy pathways, macrolipophagy and microlipophagy, are involved. Tran *et al*. [85] showed the co-localization of ATG8-marked bodies and LDs after extended N starvation in *C. reinhardtii*, while Zienkiewicz *et al*. [52] demonstrated the direct interaction between ATG8 and the LD surface protein LDSP in *N. oceanica*. Conversely, inhibiting autophagy impairs the normal degradation of LDs after nutrient resupply as shown in the *C. reinhardtii atg8* mutant [48], in the oleaginous diatom *Fistulifera solaris* [126] and in *L. incisa* [49]. Loss of PI3K activity resulted in increased lipid content in *C. reinhardtii* [127] and *N. oceanica* [52] under replete growth conditions, suggesting that autophagy negatively regulates lipid accumulation. However, this result needs to be taken cautiously as PI3K signalling regulates not only autophagy but other key energy and carbon metabolic pathways [127]. Lipophagy was shown to participate in the degradation of LDs in animals, yeast and plants [128–130] and appears to be a conserved autophagy pathway ensuring lipid homeostasis in eukaryotes. Autophagy-mediated remobilization of lipid reserves sustains cellular energy demands through lipid catabolism and membrane homeostasis during carbon starvation in *M. denticulata* [44], for the biogenesis of the photosynthetic system of *A. protothecoides* during the transition to autotrophy [81] and the reconstitution of chlorophylls and chloroplast membrane lipids after nutrient resupply in several green algae [48,49].

Interestingly, autophagy was also reported to promote TAG and LD synthesis depending on the physiological context. Pharmacological inhibition of autophagy suppressed TAG accumulation and LD formation under N and P deprivation in *C. reinhardtii* [46,131] and impaired ARA-TAG synthesis during N starvation in *L. incisa* [49]. Therefore, autophagy has a dual role in TAG and LD metabolism, promoting both their synthesis and degradation. In the plant model *A. thaliana*, basal autophagy contributes to TAG synthesis through organellar membrane lipid turnover, whereas inducible autophagy contributes to LD degradation [132]. Breaking down of membrane lipids and other carbon sources via autophagy may fuel TAG formation with necessary energy and biosynthesis precursors under nutrient deprivation in algae. Accordingly, stress-induced TAG and LD formation comes with intensive lipidome remodelling [49,133,134]. For example, starvation-induced accumulation of LDs is accompanied by enhanced breakdown of chloroplast glycerolipids and increased ARA-TAG species synthesis in *L. incisa* [49]. In summary, autophagy is emerging as a central actor of lipidome remodelling in algae, remobilizing different lipid sources (membranes, LDs) towards catabolism for energy production or to form new lipid storage structures depending on the physiological requirements of the cell.

## 4. Regulation of autophagic responses

### (a) Nutrient/energy sensing protein kinase complexes

**Figure 3.**
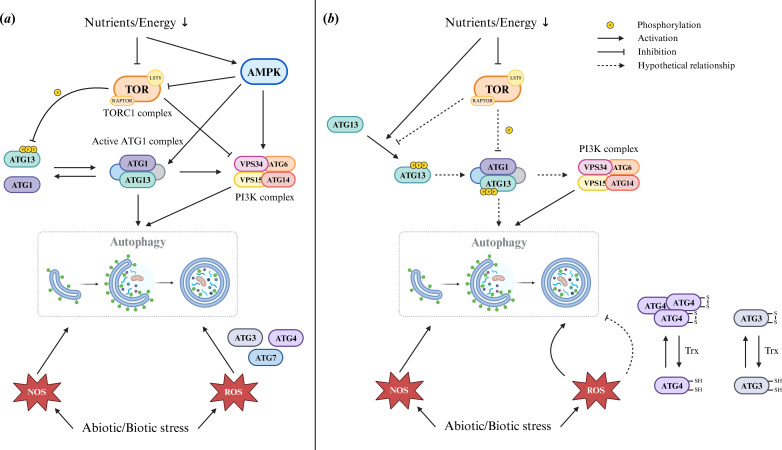
Signalling and regulation pathways of autophagy. Created with BioRender.com. (*a*) Classical model of autophagy signalling and regulation derived from yeast, mammals and plants. (*b*) Proposed model of autophagy signalling and regulation in algae.

Autophagy induction is known to be regulated by the energy status of the cell in yeast, plants and animals. The same can be said for algae given the routine induction of autophagy observed under nutrient and energy deprivation (table 1). Two decades of research have placed the target of rapamycin complex 1 (TORC1) at the centre of autophagy regulation by nutrient and energy sensing [135–137]. The TOR complex 1 includes the catalytic kinase TOR, the scaffold protein RAPTOR (regulatory associated protein of TOR), and the regulatory subunit LST8 (Lethal with Sec Thirteen 8), which stabilizes the TOR-RAPTOR interaction. All three proteins are highly conserved in eukaryotes [89,91,138], including in all algal lineages surveyed (figure 2). TORC1 acts as a negative regulator of autophagy in yeast, animals and plants, as shown by TOR activity inhibition studies [54,139,140]. In *C. reinhardtii*, rapamycin-mediated inhibition of TOR signalling triggered the accumulation and lipidation of CrATG8 and the formation of autophagosomes [43,84,85], while the haptophyte *E. huxleyi* also responded to the treatment by forming autophagic bodies [79]. In the green alga *H. lacustris*, AZD8055-mediated inhibition of TOR boosted the production of autophagic bodies during salinity stress [65]. In a similar fashion, autophagy is more strongly activated in the *C. reinhardtii lst8* mutant which shows reduced TOR activity under P deprivation [141]. To our knowledge, the effect of TOR inhibition on autophagy induction has not been tested on other prominent algal groups such as diatoms and dinoflagellates. However, TOR inhibition was shown to trigger autophagy in the stramenopile oomycete *Phytophthora sojae* [93] and in *Trypanosoma* parasites (Discoba) [62,142]. Thus, TORC1 function as a negative regulator of autophagy appears to be a common eukaryotic feature. Taken together, these observations suggest that autophagy is under the control of the rapamycin-sensitive TOR signalling pathway in algae as well (figure 3).

When active under nutrient-rich conditions, TORC1 blocks autophagy induction by phosphorylating the ATG1-activator ATG13, preventing their association, whereas ATG13 is dephosphorylated upon TOR inactivation in nutrient-poor conditions [143–146]. Whether these proteins perform a similar function in algae is unknown and should be further addressed. CrATG1 can be phosphorylated on its T802 or S803 residue; a non-statistically significant increase in this phosphopeptide abundance was measured in cultures treated with the TOR inhibitors AZD8055, Torin1 and rapamycin [147]. ATG13 phosphorylation was found to increase during N depletion and to drop after N resupply [148], showing opposite results to the classical yeast and mammalian model in which ATG13 loses its phosphorylation under TORC1 inhibition.

In addition to TORC1, autophagy is regulated by several other kinases that respond to energy status, nutrient availability and environmental stresses. In particular, the evolutionarily conserved AMPK (AMP-activated protein kinase) complex positively regulates autophagy during carbon starvation by inhibiting TORC1 and activating ATG6 and ATG1 in mammals and yeast in response to a high AMP:ATP ratio [135,137]. Its plant ortholog SnRK1 (Snf1-RELATED PROTEIN KINASE 1) performs a similar function in response to energy or nutrient deficiency [136]. Whether these central signalling kinase complexes also mediate autophagy induction in algae remains to be determined.

### (b) Oxidative stress and signalling

ROS are naturally produced as by-products of normal aerobic metabolism in cell compartments such as chloroplasts, mitochondria and peroxisomes [149]. By interfering with normal cellular metabolism and homeostasis, most abiotic and biotic stressors exacerbate the production of intracellular ROS [149,150] (table 1). Excess accumulation of ROS causes various types of cellular injuries, including damage to proteins, lipids and DNA by oxidation, rendering protein complexes and organelles dysfunctional, which need to be removed by cellular degradation mechanisms including autophagy.

ROS accumulation, induced by H_2_O_2_ or methyl viologen treatment, directly activates an autophagic response in *C. reinhardtii* [43,59]. *C. reinhardtii* mutants deficient in photoprotective pigments (e.g. carotenoids, α-carotene, lutein and loroxanthin) and in non-photochemical quenching (NPQ) showed constitutive induction of autophagy, which was aggravated when exposed to light [59]. Carotenoids prevent photo-oxidative damage through quenching of excited chlorophyll and oxygen intermediates and by dissipating excess energy as heat through NPQ [151]. Given this critical role, it is likely that the observed activation of autophagy in carotenoid and NPQ-deficient mutants is directly triggered by oxidative stress in the chloroplast. Likewise, impaired starch biosynthesis in the sta6 mutant led to constitutive high levels of ROS and CrATG8 accumulation as compared to wild type cells [73]. Furthermore, treatment with antioxidants suppressed the induction of autophagy markers in cells subjected to excess Ni^2+^ and ER stress [67,75], showing a causal effect of ROS accumulation and redox imbalance on autophagy activation.

Animal and plant cells actively produce ROS when sensing stress to initiate signalling and acclimation responses using specialized enzymatic complexes such as plasma-membrane localized NADPH oxidases [152]. Their inhibition prevents the accumulation of lipidated CrATG8 in autophagy-inducing conditions [59], demonstrating that enzymatic ROS production can elicit autophagy in stress conditions in the green algae model.

Taken together, these studies suggest that natural and enzymatic ROS accumulation is a trigger for the activation of autophagy in response to abiotic and biotic stresses in algae (figure 3). If excess ROS causes irreversible oxidative damages and activates signalling pathways promoting cell death, it has now been undoubtedly established that at low levels, ROS and their by-products can function as specific secondary messengers in pro-survival and adaptation signalling cascades [153,154].

Changes in the intracellular redox status are signalled through oxidative modification of proteins. As such, protein thiols play a crucial role in redox signalling as redox-sensitive Cys residues undergo a diverse spectrum of reversible posttranslational redox modifications, modifying their activity or their potential to interact with other proteins [153]. Such mechanisms participate in the regulation of autophagic activity. For instance, mammalian and plant ATG4 proteolytic activity is reversibly inhibited by oxidation [19,155]. In a similar fashion, yeast ATG4 activity is inhibited by the formation of a disulfide bond, whose reduction is directly catalyzed by the oxidoreductase thioredoxin Trx [156]. Perez-Perez *et al.* [108] showed that as in yeast, CrATG4 activity is regulated by the oxidoreduction of a single disulfide bond, involving the NADPH/NTR/TRXh1 system. Reduced monomeric CrATG4 actively cleaves CrATG8 while oxidized inactive ScATG4 and CrATG4 accumulate and form aggregates in autophagy-inducing stress conditions [108]. It is hypothesized that ATG4 cleaves newly synthesized ATG8 proteins constitutively in normal conditions and is inactivated under oxidative stress conditions, to prevent de-lipidation of ATG8 at the site of autophagosome formation [108].

Other core autophagy proteins are redox-regulated. ATG3 and ATG7 can be inactivated by oxidation at active site thiols in animal cells, preventing LC3/ATG8 lipidation and autophagosome formation [157]. As for ATG4, the redox-regulation of ATG3 appears to be conserved. ATG3 enzymes from both *C. reinhardtii* and *S. cerevisiae* are also rendered inactive by redox post-translational modifications under oxidative conditions [60]. ATG3 and ATG7 form a stable thio-ester covalent bond with ATG8 under normal conditions. This bond becomes transient in autophagy-inducing conditions due to ATG3 and ATG7 increased conjugase activity, rendering the enzymes sensitive to oxidation on those bond-forming thiol residues [157]. Redox inactivation of key ATG proteins under oxidative conditions may serve as a negative feedback mechanism preventing the over-activation of autophagy, as excess autophagy can lead to cell death by irreversibly compromising cellular integrity [157]. It could also be a mechanism to turn off autophagy under too high oxidation state to the benefit of pro-cell death mechanisms.

Nitric oxide (NO) is a short-lived gas that acts as a biological messenger in the regulation of a multitude of physiological processes and stress responses in animals and plants [63,158], notably activating or repressing autophagy depending on cell type, stress intensity and interaction with ROS signalling [159–162]. High light stress induces a NO burst in *C. reinhardtii* cells within a few hours, which was shown to partake in autophagy induction [61], suggesting that NO acts as a messenger in the regulation of algal autophagy too.

Rather than acting as independent pathways, TOR and ROS signalling appear to engage in intricate cross-talk in the regulation of algal autophagy induction. Nutrient starvation increases ROS production [44,163–166] and thus may trigger ROS signalling in parallel to TOR inhibition. Moreover, inhibition of TOR leads to reversible oxidation of numerous cysteine residues within 60 min in *C. reinhardtii* [167], although redox modification of ATG proteins was not reported. Therefore, both pathways may be complementary to activate autophagy during stress. The fact that ROS accumulation can be essential for autophagy induction during starvation in mammalian cells [19] and plants [21] support this hypothesis.

## 5. Conclusions and perspectives

Research on autophagy has made significant progress in land plants over the last two decades; however, it is still overlooked in other photosynthetic organisms. Developing fundamental and applied research on autophagy in algae holds significant importance for several reasons.

Marine ecosystems are never at a steady state because environmental conditions are dynamic, both in time and space, particularly regarding nutrient availability. Despite that, algae show exceptional adaptation and resilience, allowing them to thrive in these dynamic conditions by finely tuning their metabolism even in nutrient-limiting conditions, which is the norm in the huge oligotrophic regions of the global ocean. Mounting evidence suggests that the universally conserved mechanisms of autophagy are routinely recruited by distant algal lineages to recycle cellular components and molecules under changing environmental conditions. These responses may hold a particular importance in the acclimation to polar regions where algae are able to adjust to drastic season-long changes in conditions, in particular in light availability. For instance, autophagy induction has been proposed to ensure basal nutrient and energy homeostasis in the quiescent diatom *F. cylindrus* overwintering the long polar night [58].

Autophagic processes might be especially important during algal bloom events during which a dynamic succession of species is usually observed as a consequence of the availability of essential nutrients required for different groups, e.g. silicate for diatoms. An interplay between autophagy and programmed cell death signalling pathways is likely to govern cell fate decisions during active blooms, balancing population growth and collapse. Moreover, autophagic mechanisms can be hijacked by viruses to enforce lytic cycles and bloom demise, as observed in the coccolithophore *E. huxleyi* [79]. Viruses are estimated to kill about 20% of all ocean microbes each day [168], thus highlighting another likely role for autophagy in natural populations of algae. Therefore, there is a need to explore the role of autophagy in field studies, using for instance metatranscriptomic approaches to estimate the expression of ATG genes, a method that has been successfully applied during a bloom succession of diatoms and dinoflagellates [169], supporting their role in nutrient recycling under limiting conditions in the predominant species.

Better understanding the mechanisms of autophagy in algae is also of interest for industrial purposes, since these are tightly linked with lipid metabolism—both anabolism and degradation—and may allow for the development of new efficient strategies to achieve higher biomass yields and productivity for biofuel production, nutraceuticals and the feed sector. All these above points require extending the availability of autophagy markers and molecular tools, well established in *C. reinhardtii* (reviewed in [170]), to other algal groups, especially diatoms as genetic transformation is readily available in several species.

Therefore, we propose the following axes as priority for future algae autophagy research development:

— probing the importance and distribution of autophagic processes in stress acclimation in ocean field studies by including autophagy markers in metatranscriptomic approaches;
— exploring the cross-talk between autophagy and cell death, especially in the context of environmental stress and bloom dynamics;
— investigating the cross-talk between lipid metabolism and autophagy for the development of efficient strategies to achieve higher algal biomass and better microalgal lipid productivity;
— extending the availability of markers and genetic tools to measure the autophagic flux to other algal lineages, especially photosynthetic stramenopiles where genetic transformation is readily available in several taxa.

Overall, continued exploration of autophagy in algae promises to offer both fundamental biological insights and practical applications with potential implications for various fields, ranging from environmental science to biotechnology.

## Data Availability

New data presented in this review paper are available as electronic supplementary materials [171].
